# Lord Brougham on Partial Insanity

**Published:** 1849-04-01

**Authors:** 


### ON PARTIAL INSANITY.

BY LORD BROUGHAM.

WE copy the following observations on Partial Insanity from our able cotemporary, *The Jurist*. They arose out of an appeal, heard before the Privy Council, from the sentence of the Court of Arches, refusing probate to the will of Sarah Gibson, published, or purporting to have been published, on the 1st of March, 1834. It will be perceived that his lordship, in delivering judgment, repudiates the idea of partial insanity, maintaining the *unity* and *indivisibility of the mind.* He also argues, that if the intellect be unsound on one point—the unsoundness at all times existing—it is erroneous to suppose the mind to be in a really sane state on other subjects. The will of such a person, apparently ever so rational and proper, is legally void. In the eye of the law, a delusion is defined to be—a belief in things as realities, which exist only in the imagination of the individual. The incapacity to struggle against a delusion constitutes unsoundness of mind. To constitute a "lucid interval," the party must freely and voluntarily, and without the design of pretending sanity, confess his delusion. After making a few preliminary observations, Lord Brougham observes—

"The principles which must govern a case of this description are sufficiently clear, and they may be regarded as well settled by the current of former decisions. Indeed, they flow easily, from considering the nature of the inquiry in which such cases engage us. The question being, whether the will was duly made by a person of sound mind or not, our inquiry, of course, is whether or not the party possessed his faculties, and possessed them in a healthy state. His mental powers may be still subsisting, no disease may have taken them away, and yet they may have been affected with disease, and thus may not have entitled their possessor to the appellation of a person whose mind was sound. Again, the disease affecting them may have been more or less general—it may have extended over a quarter, or a less portion of the understanding; or rather we ought to say, that it may have affected more, or it may have affected fewer, of the mental faculties: for we must keep always in view that which the inaccuracy of ordinary language inclines us to forget, that the mind is one and indivisible; that, when we speak of its different powers or faculties, as memory, imagination, consciousness, we speak metaphorically, likening the mind to the body, as if it had members or compartments; whereas, in all accuracy of speech, we mean to speak of the mind acting variously—that is, remembering, fancying, reflecting—the same mind, in all these operations, being the agent. We therefore cannot, in any correctness of language, speak of general or partial insanity; but we may, most accurately, speak of the mind exerting itself in consciousness without cloud or imperfection, but being morbid when it fancies: and so its owner may have a diseased imagination; or the imagination may not be diseased, and yet the memory may be impaired, and the owner be said to have lost his memory. In these cases, we do not mean that the mind has one faculty, as consciousness, sound, while another, as memory, or imagination, is diseased; but that the mind is sound when reflecting on its own operations, and diseased when exercising the combination, termed imagining, or casting the retrospect, called recollection. This view of the subject, though apparently simple, and almost too unquestionable to require, or even justify, a formal statement, is of considerable importance when we come to examine cases of what are called, incorrectly, partial insanity, which would be better described by the phrase "insanity" or "unsoundness," always existing, though only occasionally manifest. Nothing is more certain than the existence of mental disease of this description. Nay, by far the greater number of morbid cases belong to this class. They have acquired a name—the disease called familiarly, as well as by physicians, "monomania," on the supposition of its being confined, which it rarely is, to a single faculty, or exercise of the mind, which is sound, to all appearances, upon all subjects save one or two, and on these he shall be subject to delusions, mistaking for realities the suggestions of his imaginations. The disease here is said to be in the imagination,—that is, the patient's mind is morbid, or unsound, when it imagines; healthy and sound when it remembers. Nay, he may be of unsound mind when his imagination is employed, on some subjects, in making combinations; and sound when making others, or making one single kind of combination. Thus, he may not believe all his fancies to be realities, but only some, or one; of such a person we morally predicate, that he is of unsound mind only upon certain points. I have qualified the propositions thus on purpose, because if the being, or essence, which we term the mind, is unsound on one subject, provided that unsoundness is at all times existing upon that subject, it is quite erroneous to suppose such a mind really sound on other subjects. It is only sound in appearance; for, if the subject of the delusion be presented to it, the unsoundness which is manifested, by believing in the suggestions of fancy as if they were realities, would break out; consequently, it is as absurd to speak of this as a really sound mind—a mind sound when the subject of the delusion is not presented—as it would be to say that a person had not the gout, because, his attention being diverted from the pain by some more powerful sensation, by which the person was affected, he for the moment was unconscious of his visitation. It follows from hence that no confidence can be placed in the acts, or in any act, of a diseased mind, however apparently rational that act may appear to be, or may in reality be. The act in question may be exactly such as a person without mental infirmity might do. But there is this difference between the two cases: the person uniformly and always of sound mind could not, at the moment of the act done, be the prey of morbid delusion, whatever subject was presented to his mind; whereas the person called partially insane—that is to say, sometimes appearing to be of sound, sometimes of unsound mind—would inevitably show his subjection to the disease the instant its topic was suggested. Therefore we can, with perfect confidence, rely on the act done by the former, because we are sure that no lurking insanity, no particular, or partial, or occasional delusion does mingle itself with the person's act, and materially affect it. But we never can rely on the act, however rational in appearance, done by the latter, because we have no security that the lurking delusion, the real unsoundness, does not mingle itself with, or occasion, the act. We are wrong in speaking of partial unsoundness; we are less incorrect in speaking of occasional unsoundness. We should say that the unsoundness always exists; but it requires a peculiar topic, else it lurks and appears not. But the malady is there; and as the mind is one and the same, it is really diseased, while apparently sound; and really its acts, whatever appearance they may put on, are only the acts of a morbid or unsound mind. Unless this reasoning be well founded, we cannot account for the unanimity with which men have always agreed in regarding, as the acts of an insane mind, those acts, to all appearance rational, which a person does who labours under delusions of a plainly extravagant nature, though there is nothing in the act done, and nothing in the conduct of the party while doing it, at all connected with the morbid fancies. If these fancies only affect the party now and then—if for some months he is free from them, labouring under them at other times—then his acts, apparently rational, would not be regarded as those of a person mentally diseased. But, if we were convinced that, at the time of doing the acts, the delusion continued, and was only latent by reason of the mind not having been pointed to its subject, and would have instantly shown itself had that subject been presented, then the act is at once regarded as that of a madman. Thus, there have been many cases of persons labouring under the delusion that they were other than themselves: some have believed themselves deceased emperors or conquerors; others, supernatural beings. Suppose one, who believed himself the Emperor of Germany, and on all other subjects was apparently of sound mind, did any act requiring mind, memory, and understanding—suppose he made his will, and either did not sign it before signing was required, or, if he did, signed with his own name, but suppose we were quite convinced, that, had any one spoken of the Germanic Diet, or proceeded to abuse the German emperor, the testator's delusion would at once break forth—then we must at once pronounce the will void, be it as efficacious and as rational in every respect as any disposition of property could be; of course, no one could propound such a will with any hopes of probate, if it happened that, while making it, the delusion had broken out, even although the instrument bore no marks of its existence at the time of its concoction. It must always be a question of evidence, on the whole facts and circumstance of the case, whether or not the morbid delusion existed at the time of the factum—that is, whether, had the subject of it been presented, the chord been struck, there would have arisen the insane discord, which is absent to all outward appearance from the chord not having been struck. The principles which have been laid down do not at all differ from those on which the Courts have acted, which text-writers have constructed, and which scientific men, both moralists and physicians, have approved. In the well-known case of *Dew* v. *Clark*—reported 3 Add. 97, but also reported, with the great advantage of the learned judge's corrections, and published separately by Dr. Haggard—we find Sir John Nicholl stating, that mere eccentricity is not enough to constitute mental unsoundness, nor great caprice, nor violence of temper, but that there must be an aberration of reason; and he adopts a definition of delusion given by the learned counsel in the cause, (now a member of this Court,) believing it well described by the expression, that it is "a belief of facts which no rational person would have believed." Perhaps, in a strictly logical view, this definition is liable to one exception, and, at least, exposed to one inaccuracy—that it gives a consequence for a definition; and it may be more strictly accurate to term "delusion" the belief of things as realities which exist only in the imagination of the patient. The frame or state of mind which indicates his incapacity to struggle against such an erroneous belief, constitutes an unsound frame of mind. Sir John Nicholl justly adds, that such delusions are generally attended with eccentricities—often with violence—very often with exaggerated suspicions and jealousies. Lord Hale lays it down, that insanity may be general, and it may be partial. "There is," says he, "a partial insanity of mind, and there is a total insanity;" and the former, he says, is expressed by the phrase "quoad hoc vel illud insanire." (1 P. C., c. 4. s. 2.) But Sir John Nicholl (and Hale does not differ) speaks of partial insanity as only that which is occasionally called forth, and not that which only exists occasionally. The authority of Lord Hale is quite consistent with the position, that the disease is always present, and not apparent by the accident of the proper chord not having been struck at the time; and Sir John Nicholl more expressly says, in explaining what he means by *occasional*, "a delusion, not called forth except under particular circumstances." In all the cases there are delusions occasionally manifested, and a state of mind incapable of mastering them. (Hagg. p. 6.) Dr. Willis, on Mental Derangement, p. 151, clearly states than men often mistake, for a lucid interval, the mere absence of the subject of delusion from the mind. He says, no madman can be said to have recovered his reason unless he freely and voluntarily confesses his delusion: to which I take leave to add, that the confession or admission must be, not only freely and voluntarily, but made without any design at the time of pretending sanity and freedom from delusion, according to the known and suspected view of the inquirer, and acting a part accordingly. There is a noted instance of the power sometimes possessed by lunatics to restrain for the moment, and for a purpose, their imagination, and conceal their delusions. It occurred in a case, tried at Guildhall, by Lord Mansfield, where one Wood, who had indicted Dr. Munro for detaining him in his madhouse, was able completely to evade all questions on his delusions, though some time before, in another indictment, tried at Westminster, he had readily fallen into them when examined. The defendant accordingly was obliged to give evidence at Guildhall of what had taken place at Westminster. (27 How. St. Tr.) If these are the principles upon which all cases of this description must be decided, there are others applicable to all cases whatsoever, but especially to such as rest upon circumstantial evidence. The burthen of the proof often shifts about in the process of the cause, accordingly as the successive steps of the inquiry, by leading to inferences decisive, until rebutted, cast on one or the other party the necessity of protecting himself from the consequences of such inferences; nor can anything be less profitable, as a guide to our ultimate judgment, than the assertion which all parties are so ready to put forward in their behalf severally, that, in the question under consideration, the proof is on the opposite side. Thus, no doubt, he who propounds a later will, undertakes to satisfy the court of probate that the testator made it and was of sound and disposing mind. But very slight proof of this, where the factum is regular, will suffice; and they who impeach the instrument must produce their proofs, should the party *actor*, the party propounding, choose to rest satisfied with his *primâ facie* case, after an issue tendered against him: in this event, the proof has shifted to the impugner, but his case may easily shift it back again. So, where any circumstances of grave suspicion arise at the outset of a case, as that a will is shown in the outset to have been made and published in a lunatic asylum, (which I have known to happen,) the burthen of proving, and very satisfactorily proving, the testator's sanity, would be so clearly on the propounding party, that no further proof would be required to impugn it. In the present case, there is a circumstance of a somewhat similar description. It is not denied that, some years after the factum—that is, in 1841, the testatrix was found a lunatic by inquisition; that she died undoubtedly insane, and that the madness was found by the jury to go back to within four years of the date of the will. This clearly made it incumbent on the party propounding to show the sanity, by much clearer proof than would have been required, had no such disease been admitted, on all hands, to have clouded her understanding towards the close of her life. But it is also to be observed, that insane delusions are very clearly shown to have taken possession of her mind previously to the date of the will; and, although the degree of disease which then existed has been made the subject of dispute, no one can pretend that there was perfect soundness of mind some few years before March, 1834. This renders it still more necessary for the court of probate to be satisfied that these delusions had ceased, and the mind recovered its healthy state before the factum. Nor is this all: the delusions, which existed at an early date, are proved to have increased after the will was made. They gathered force until it became necessary to sue out a commission; and the result of the inquisition was, that in 1838 she had become perfectly insane. Thus it becomes quite impossible to disconnect the different periods of this unhappy person's history. There is every probability that the diseased state, which commenced before the factum, continued up to its date. The likelihood is, that the delusions, of which evidence exists before and after, continued during the intermediate time, although no proofs may be obtained of the precise fact; and all the presumptions, which would otherwise have been in favour of sanity at that date, are turned the other way by these important circumstances. Hence, it is not at all a just and correct view of this case, which affirms that the presumption is in favour of the testatrix's soundness; and the proof of continued delusion is thrown upon those who deny it, merely because there is no evidence directly applicable to the date of the will. No one, who finds a person labouring under the same kind of delusions before and after a given period, can be justified in refusing his belief to their continuance during the interval, unless clear evidence be produced of their having ceased for a time, and then returned. The very great probability is, that they existed all the while, and were only not apparent, because the subject with which they were connected did not happen to be openly mentioned before others who might give evidence. The very great probability is, that the patient laboured under them all the while, although she did not openly declare her belief in them, and act or speak under that belief. Another observation remains to be offered, before proceeding to a more minute commentary on the evidence. The existence of delusions being proved, and their continuance proved or assumed at the date of the factum, so that the court is satisfied of the testatrix then labouring under their influence, it is wholly immaterial that they do not appear in the will itself. The party propounding often approached this point in argument, and repeatedly adverted to the fact—perhaps we should rather say, the assertion or the assumption—that this will betrays no marks of the alleged delusions, or generally of an unsound mind. There was a manifest disposition to lay down a rule, that no person labouring under monomania or partial insanity can be deemed intestable, unless the kind of insanity appears on the face of the will. But there was wanting the courage to lay down a position which would at once have been rejected, and must have been met by the question—'Could any court admit to probate the will of the man who said, (in the case cited by Sir John Nicholl, in *Dew* v. *Clarke*,) 'I am the Christ,' although that will bear no marks whatever of an unsound mind, still less of the dreadful delusion under which the party laboured?' It is hardly possible, on the other hand, that any will can be so framed as to rebut all presumptions of insanity arising from proved facts."

It was our intention to have made some lengthy comments on the above judgment of Lord Brougham, which press of matter compels us to defer until the next number, when the important subject of partial insanity, in its legal bearings, will be fully considered.

---

